# Phenylalanine 4-Hydroxylase Contributes to Endophytic Bacterium *Pseudomonas fluorescens*’ Melatonin Biosynthesis

**DOI:** 10.3389/fgene.2021.746392

**Published:** 2021-11-15

**Authors:** Jian Jiao, Yan Xia, Yingli Zhang, Xueli Wu, Chonghuai Liu, Jiancan Feng, Xianbo Zheng, Shangwei Song, Tuanhui Bai, Chunhui Song, Miaomiao Wang, Hongguang Pang

**Affiliations:** ^1^ College of Horticulture, Henan Agricultural University, Zhengzhou, China; ^2^ Henan Key Laboratory of Fruit and Cucurbit Biology, Zhengzhou, China; ^3^ Zhengzhou Fruit Research Institute, Chinese Academy of Agricultural Sciences, Zhengzhou, China

**Keywords:** melatonin, endophytic bacteria, phenylalanine 4-hydroxylase, aromatic amino acid hydroxylases, 5-hydroxytryptophan

## Abstract

Melatonin acts both as an antioxidant and as a growth regulatory substance in plants. *Pseudomonas fluorescens* endophytic bacterium has been shown to produce melatonin and increase plant resistance to abiotic stressors through increasing endogenous melatonin. However, in bacteria, genes are still not known to be melatonin-related. Here, we reported that the bacterial phenylalanine 4-hydroxylase (PAH) may be involved in the 5-hydroxytryptophan (5-HTP) biosynthesis and further influenced the subsequent production of melatonin in *P. fluorescens*. The purified PAH protein of *P. fluorescens* not only hydroxylated phenylalanine but also exhibited l-tryptophan (l-Trp) hydroxylase activity by converting l-Trp to 5-HTP *in vitro*. However, bacterial PAH displayed lower activity and affinity for l-Trp than l-phenylalanine. Notably, the PAH deletion of *P. fluorescens* blocked melatonin production by causing a significant decline in 5-HTP levels and thus decreased the resistance to abiotic stress. Overall, this study revealed a possible role for bacterial PAH in controlling 5-HTP and melatonin biosynthesis in bacteria, and expanded the current knowledge of melatonin production in microorganisms.

## Introduction

Melatonin is a tryptophan-derived natural product and now recognized as a ubiquitous biomolecule synthesized in almost all species of animals, plants, and unicellular algae (Balzer and Hardeland, 1996; Hardeland and Poeggeler, 2003; Rodriguez-Naranjo et al., 2012; Arnao and Hernández-Ruiz, 2015). Melatonin is presumed to be an ancient molecule that appeared in primitive photosynthetic bacteria, and it originally functions as a free radical scavenger and antioxidant (Hardeland et al., 1995; Tan et al., 2015). The antioxidative function of melatonin is thought to be intrinsic and evolutionarily conserved in almost all organisms, including bacteria. During evolution, melatonin has been adopted by multicellular organisms to perform many other biological functions, such as acting as a signaling molecule to modulate mood, sleep, seasonal reproduction, and circadian rhythms in vertebrates (Hardeland, 2008; Pandi-Perumal et al., 2008; Reiter et al., 2009). In plants, melatonin has been reported to be involved in seed germination (Zhang et al., 2013; Zhang et al., 2014), flowering (Kolá et al., 2003), fruit maturation (Sun et al., 2016), root development (Pelagio-Flores et al., 2012), delaying chlorophyll degradation, leaf senescence (Zhang et al., 2016a), and tolerance against biotic and abiotic stress (Arnao and Hernández-Ruiz, 2010; Nawaz et al., 2016).

As shown in Supplementary Figure S1, the classic melatonin biosynthesis pathway in animals starts with tryptophan and involves four sequential reactions: tryptophan → 5-HTP → serotonin → *N*-acetylserotonin → melatonin. However, melatonin synthesis involves multiple pathways and can be regulated by up to six enzymes in plants (Back et al., 2016). Among the two enzymatic steps involved in the conversion of tryptophan to serotonin, 5-HTP is the first intermediate in animals catalyzed by tryptophan 5-hydroxylase (TPH) (Fitzpatrick, 2003); however, plants initially decarboxylate tryptophan *via* tryptophan decarboxylase to form tryptamine (Byeon et al., 2014). Currently, plants are also believed to catalyze tryptophan to 5-HTP, whereas the putative gene encoding TPH enzyme has yet to be identified (Back et al., 2016). The melatonin synthesis machinery in eukaryotes is presumed to have been inherited from bacteria *via* endosymbiosis (Tan et al., 2015). This is because primitive bacteria may be the precursors of mitochondria and chloroplasts, and the two organelles were proved to be the dominant sites for melatonin production in animals and plants (Tan et al., 2013). However, only scattered reports exist on the occurrence of melatonin in bacterial microbes, such as aerobic photosynthetic cyanobacteria and proteobacteria (Manchester et al., 1995; Hardeland and Poeggeler, 2003; Byeon et al., 2013) or recombinant *E. coli* (Byeon and Back, 2016). The mechanism of bacterial melatonin biosynthesis is still unknown.

Our group previously reported that some endophytic bacteria isolated from grape roots could produce melatonin *in vitro* (Jiao et al., 2016). The melatonin-producing endophytic bacterium of *Pseudomonas fluorescens* RG11 was proved to biosynthesize 5-HTP but not tryptamine (Ma et al., 2017), suggesting that TPH may be present in this strain. However, in the *P. fluorescens* genome sequences posted in GenBank, the corresponding TPH-encoding genes that had been identified are missing.

TPH is a member of the pterin-dependent aromatic amino acid hydroxylase (AAAH) enzyme family that also includes phenylalanine 4-hydroxylases/monooxygenase (PAH) and tyrosine 3-hydroxylase (TH) (Kappock and Caradonna, 2016). These three enzymes are named after their specific amino acid substrates and show high sequence identity and similar secondary structure and catalytic mechanism (Flydal and Martinez, 2013). Notably, AAAHs are widely distributed among bacterial species, but a majority of them were identified as PAH with the potential to hydroxylate l-Phe to yield tyrosine (Kaufman, 1993; Fitzpatrick, 2000). Moreover, the PAH of *Chromobacterium violaceum* and *P. fluorescens* had been reported to use l-Trp as a substrate to form 5-HTP, albeit with much lower efficiency than l-Phe (Kino et al., 2009). Thus, the bacterial PAHs might be responsible for the conversion of l-Trp to 5-HTP and may play a pivotal role in melatonin biosynthesis in *P. fluorescens* RG11. However, this putative melatonin-biosynthesizing mechanism needs to be tested experimentally.

To prove the hypothesis, the PAH-encoding gene from *P. fluorescens* RG11 was cloned and expressed the PAH protein in *Escherichia coli* for functional identification. A mutant strain of *P. fluorescens* lacking functional PAH was also generated to investigate the effect of PAH on bacterial melatonin biosynthesis. Taken together, this work demonstrated a correlation between bacterial PAH and melatonin biosynthesis, and it expands the available information on the role of melatonin in microorganism.

## Materials and Methods

### Bacterial Strains, Plasmids, and Growth Conditions

The strains and plasmids used in this study are listed in Supplementary Table S1. The melatonin-synthesizing endophytic bacterium *P. fluorescens* RG11, previously isolated from the roots of *V. vinifera* cv. Red Globe (Ma et al., 2017), was aerobically grown in beef extract-peptone (BP) liquid medium (3 g L^−1^ beef extract, 10 g L^−1^ tryptone, 5 g L^−1^ NaCl; pH 7.4) at 28°C. *E. coli* DH5α and BL21 (DE3) were used as the host for PAH gene cloning and expression, respectively. The plasmid pMD18-T (TaKaRa, Dalian, China) was used as a vector for gene cloning, and pET-30a (+) (Novagen, Darmstadt, Germany) was used for gene expression. *E. coli* strains were grown in Luria-Bertani (LB) medium containing 10 g/L tryptone, 5 g/L yeast extract, and 10 g/L NaCl at 37°C. Purified agar (1.0%; Pronadisa, Madrid, Spain) was added for solid LB medium. When required, the medium was supplemented with kanamycin or ampicillin at final concentrations of 50 and 100 mg/L, respectively.

### Phenylalanine 4-Hydroxylase Gene Cloning, Phylogenetic Tree Construction, and Sequence Alignment

Genomic DNA of *P. fluorescens* RG11 was extracted with the EZNA Bacterial DNA kit (Omega Bio-Tek, Norcross, GA, United States) as recommended by the manufacturer. The full-length gene encoding PAH was amplified with the primers pah-f (5′-ATG​AAG​CAG​ACG​CAA​TAC​GTG-3′) and pah-r (5′-TCA​CGC​GGC​TTT​CGG​TTT​T-3′) based on sequence information (GenBank accession no. NC_007,492). The PCR products were cloned into the pMD18-T vector for sequencing. Plasmids were routinely isolated from *E. coli* DH5α using the TIANprep Rapid Mini Plasmid Kit (Tiangen, Beijing, China).

Protein sequences were obtained by querying the NCBI protein database (https://www.ncbi.nlm.nih.gov/protein/) for “phenylalanine 4-hydroxylase/monooxygenase,” “tyrosine 3-hydroxylase,” and “tryptophan 5-hydroxylase” as the keywords. In total, 38 AAAH amino acid sequences derived from animals (*Homo sapiens*, *Mus musculus*, *Gallus gallus*, and *Bos taurus*) and prokaryotic bacteria were randomly selected, and a phylogenetic tree was constructed with the neighbor-joining method using MEGA X software (Kumar et al., 2018). Some TPHs and PAHs were aligned using ClustalX version 2.1, and the figure was created using ESPript3.0 (http://espript.ibcp.fr/ESPript/ESPript/) (Robert and Gouet, 2014).

### Detection of PfPAH Gene Expression

Total RNA was isolated from *P. fluorescens* RG11 cultured in BP liquid medium using an E.Z.N.A. Bacterial RNA kit (OMEGA Bio-Tek, United States) according to the manufacturer’s instructions. Following quantification with the NanoDrop ND-1000 (Thermo Scientific, United States), RNA was reverse transcribed into cDNA with the HiScript III 1st Strand cDNA Synthesis Kit with gDNA wiper (Vazyme, Nanjing, China) and used as a template for RT-PCR. Primers pah-f/pah-r were used to assess transcription of the PAH-encoding gene by amplifying the full-length sequence. To eliminate contributions from contaminating DNA in the DNase-treated samples, control experiments were performed in the absence of reverse transcriptase.

### Phenylalanine 4-Hydroxylase Overexpression and Protein Purification

The prokaryotic expression vector pET-30a (+) was used for C-terminal His-tagged expression. The full-length *phhA* gene, encoding the *P. fluorescens*’ PAH, was PCR-amplified with the following primers: 5′-CAT​ACAT​ATGAAG​CAG​ACG​CAA​TAC​GTG-3′ (forward primer; the *Nde*I site is underlined) and 5′-GTGCTC​GAGCGC​GGC​TTT​CGG​TTT​T-3′ (reverse primer; the *Xho*I site is underlined). The amplified products were then purified, digested with *Nde*I and *Xho*I, gel-purified, and ligated into pET-30a. The resulting recombinant plasmid, named pET-30a-*phhA*, was transformed into *E. coli* BL21 (DE3) competent cells.

The *E. coli* BL21-pET-30a-*phhA* strain was cultured in LB medium supplemented with 50 μg/ml kanamycin at 37°C. When the OD_600_ reached approximately 0.6, isopropyl-β-d-1-thiogalactopyranoside (IPTG, Sigma, St. Louis, MO, United States) was added to a final concentration of 1 mM to induce protein expression. After a 4-h incubation at 24°C with shaking at 220 rpm, cells were collected by centrifugation (5,000 × *g* for 12 min at 4°C), re-suspended in lysis buffer (50 mM Na-phosphate, 300 mM NaCl, 10 mM imidazole, pH 8.0; 3 ml per 1 g of cell pellets) supplemented with protease inhibitors (Beyotime, China), and then sonicated by ultrasonication (300 W, ice). The lysates were subsequently centrifuged at 12,000 × *g* for 20 min at 4°C, and the supernatants were added to a Ni-NTA agarose column (QIAGEN China Co., Ltd., Shanghai). After washing with wash buffer (50 mM Na-phosphate, 300 mM NaCl, 20 mM imidazole, pH 8.0), the bound PfPAH was eluted by the wash buffer supplemented with 250 mM imidazole. The proteins were analyzed by SDS-PAGE, and their concentrations were estimated using a BCA Protein Assay Kit (Tiangen, Beijing, China).

### Measurement of Hydroxylation Activity Towards l-tryptophan and l-phenylalanine

The enzymatic activity of purified PfPAH-(His)_6_ was measured at a standard concentration of reactants (0.5 ml total) in 50 mM NaOH-HEPES buffer (pH 7.5) containing 500 U of catalase, 5 mM dithiothreitol, 0.5 mM tetrahydrobiopterin (BH_4_), and 100 μM FeSO_4_, as described previously (Kino et al., 2009) with some modifications. Briefly, the reactions were initiated by adding 0.25 μg of protein and variable concentrations of substrates, including l-Trp (0.4–20 mM) or l-Phe (0.1–2 mM). After incubation for 10 min at 35°C, the reaction was stopped by the addition of 0.5 ml of methanol. The amount of synthesized 5-HTP or l-tyrosine was measured using UPLC-MS/MS as described in our previous study (Jiao et al., 2016) with the quantification transitions of *m/z* ^+^ 182→136 and *m/z* ^+^ 221→204. The kinetic parameters for both substrates were calculated using Michaelis–Menten analysis. The effect of temperature on l-Trp hydroxylase activity for PfPAH was determined by pre-incubating the reaction mixture at temperatures from 10 to 55°C for 5 min. The reactions were initiated by adding 20 mM L-Trp into the standard mixture above and incubating for 10 min at the same temperature. All experiments were performed in triplicate.

### Generation of Phenylalanine 4-Hydroxylase Deletion Mutants

As the first step toward obtaining PAH deletion mutants (∆*phhA*), fragments upstream and downstream of *phhA* were amplified using primer pairs *phhA*-F1/*phhA*-R1 and *phhA*-F2/*phhA*-R2, respectively (sequences shown in Supplementary Table S2). The two PCR products were gel-purified and ligated using splicing by overlap extension PCR with the primer pairs *phhA*-F1/*phhA*-R2. The resulting fragment was cloned into the pMD18-T vector and then sub-cloned into the suicide vector pK18*mobsacB* (Schäfer et al., 1994) to produce pK18-∆*phhA*. Gene replacement was performed through double homologous recombination. In brief, pK18-∆*phhA* was introduced into the *P. fluorescens* RG11 strain by triparental mating using a helper strain *E. coli* DH5α containing the plasmid pRK600 (Finan et al., 1986). BP selection medium containing 1.5% agar and 50 μg/ml kanamycin was used to screen the first recombination events. The second crossover event was then selected by incubating kanamycin-resistant clones into a liquid medium supplemented with kanamycin for 24 h, and then plating onto BP plates containing 10% (w/v) sucrose. Double crossover recombinants were confirmed by PCR and sequenced with *phhA*-F1/*phhA*-R2 primers.

### Microbial Cultivation With ^15^N Double-Labeled l-Tryptophan and UPLC-MS/MS Analysis

Wild-type and ∆*phhA* mutant RG11 strains were individually transferred to 20 ml of BP liquid medium and cultivated for 8–12 h at 28°C to a final OD_600_ of approximately 1.0. After harvesting at 6,000 × *g* for 10 min, the cells were re-suspended to 1 × 10^8^ cells ml^−1^ in 0.9% sterilized saline solution using a Petroff-Hausser counting chamber (Hausser Scientific Company, Horsham, PA, United States). The resulting bacterial inocula (1 ml) were added to individual 100-ml brown bottles containing 50 ml of BP liquid medium with 500 mg L^−1 15^N double-labeled l-Trp (Cambridge Isotope Laboratories, Andover, MA, United States). The cultures were incubated in a rotary shaker at 32°C with 160 rpm agitation in the dark under aerobic conditions. Three replicates were performed for each treatment.

Samples were obtained every 6 h for viable cell counts using the plate counting method after sufficient dilutions. The bacterial culture (1 ml) was diluted 1:1 with methanol and ultrasonicated (300 W, ice water) for 10 min to assist the extraction of melatonin. The resulting mixture was filtered using a 0.22-μm filter and then used to quantify ^15^N-tryptamine, ^15^N-5-HTP, ^15^N-serotonin, ^15^N-5-methoxytryptamine, ^15^N-*N*-acetylserotonin, and ^15^N-melatonin with UPLC-MS/MS as described in our previous study (Ma et al., 2017). All non-labeled standards were purchased from Sigma-Aldrich (St. Louis, MO, United States). The experiment was repeated in triplicate. The Student’s *t*-test was used to identify significant differences in melatonin levels and its precursors between wild-type and ∆*phhA* strains.

### The Abiotic Stress Tolerance of ∆*phhA* Mutant and Wild-Type of *P. fluorescens*

The strain of ∆*phhA* mutant and wild-type of *P. fluorescens* were routinely transferred to LB medium and cultivated for 12–16 h at 37°C with 150 rpm of agitation to the final desired bacterial concentration of 1.5 × 10^8^ CFU ml^−1^ (OD_600_ of about 1.0). The cultures were harvested at this stage and used as the inoculum in the following experiments.

To investigate the abiotic stress tolerance of wild-type *P. fluorescens* toward NaCl, drought, and heat, 1 ml of inoculum was inoculated into 150 ml of LB and then treated with different conditions: (i) control (37°C); (ii) 400, 600, 1,000, 1,500, and 2,000 mM NaCl at 37°C; (iii) drought [20%, 30%, 40%, 50%, and 60% polyethylene glycol 6000 (PEG6000) at 37°C]; and (iv) high temperature (44°C, 48°C, 52°C, and 56°C). Then, the flasks were incubated in a thermostat shaker set to 150 rpm. Cultures were collected after 12 h, and viable cells (log10 CFU/ml) were counted by serial dilution plating on LB plates in triplicate. The experiment was repeated three times.

To test the effect of PAH on bacterial abiotic stress resistance, the cultures of ∆*phhA* mutant and wild-type of *P. fluorescens* were treated with NaCl (1.5 M), drought (50% PEG6000), or high temperature (52°C). An aliquot of 0.5 ml was taken at 1-h intervals, diluted in PBS buffer, and plated on LB plates in triplicate. The number of colonies appearing on the plates after 24 h of incubation at 37°C were counted.

## Results

### Identification of the Phenylalanine 4-Hydroxylase Gene in *P. fluorescens* RG11 and Sequence Analysis

**FIGURE 1:**
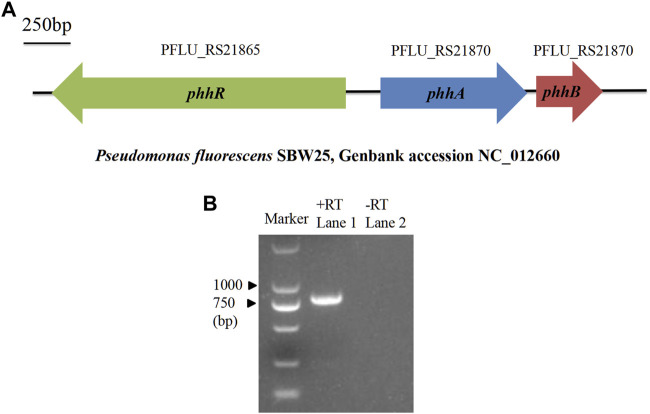
Location and expression of *phhA*. **(A)** Description of the location of *phhA* and its neighboring genes in the *P. fluorescens* genome. The horizontal arrows indicate the genes’ orientation and relative size. *phhR* and *phhB* are responsible for encoding sigma-54-dependent phenylalanine hydroxylase transcriptional regulator and pterin-4α-carbinolamine dehydratase, respectively. **(B)** Reverse transcription (RT)-PCR analysis of the *phhA* expression in the RG11 strain after culturing in BP liquid medium. Lane 1, RT-PCR with primers designed for full-length *phhA* (792 bp)*,* and the cDNA was used as the template; lane 2, the same process as in the assay in lane 1, but used mRNA as a template without reverse transcription.

The *phhA* gene, encoding a PAH ortholog, was found in genomes of most *P. fluorescens* strains by querying “phenylalanine 4-monooxygenase” in the NCBI nucleotide database. In the previously sequenced chromosomal DNA of *P. fluorescens* SBW25 (NC_012,660; Figure 1A), the *phhA* gene had been designated as the ORF PFLU_RS21870, and mapped between the ORFs of *phhR* and *phhB*. RT-PCR analysis confirmed that *phhA* was expressed in the RG11 strain after culturing in BP liquid medium (Figure 1B).

**FIGURE 2:**
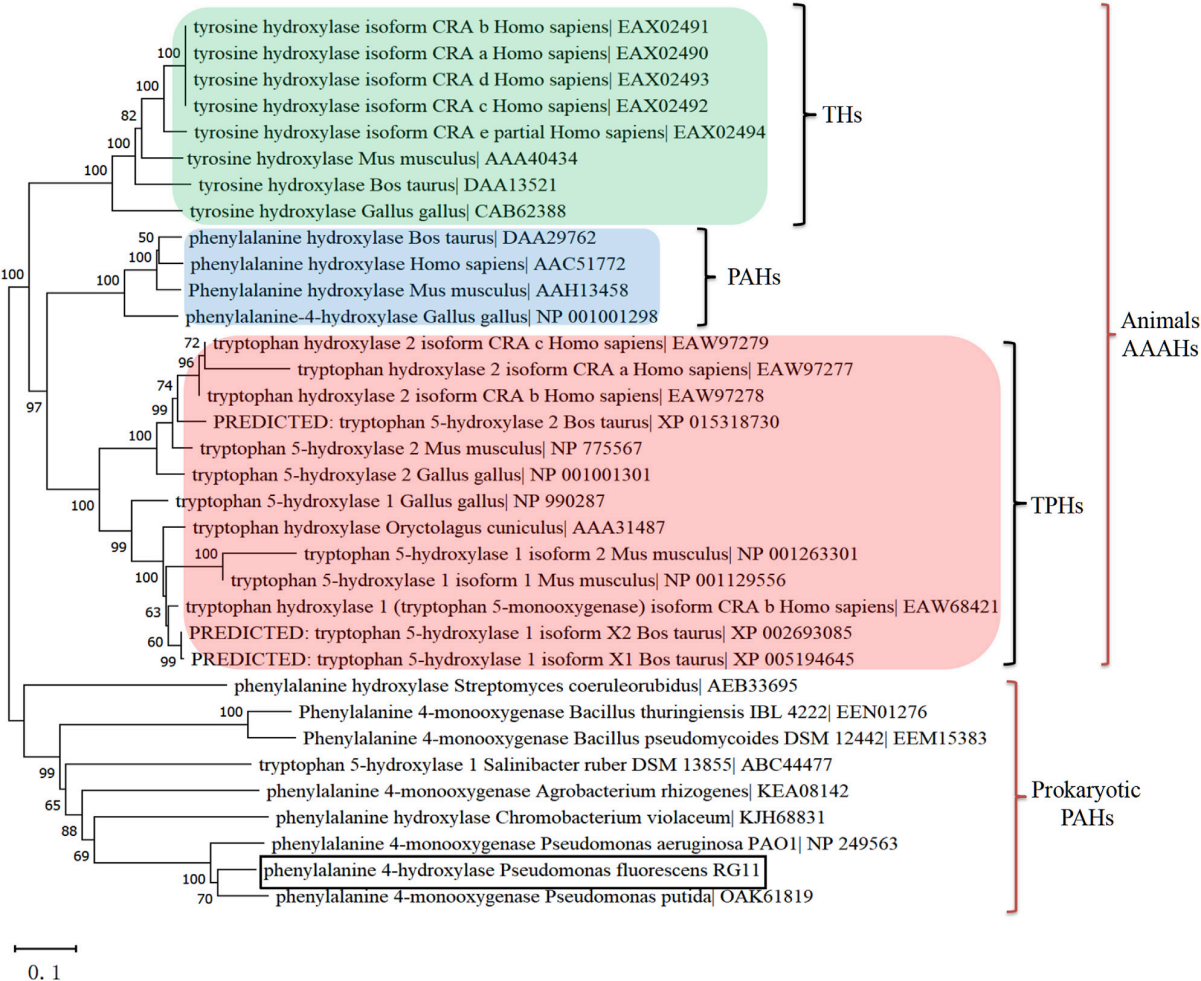
Rooted phylogenetic tree based on prokaryotic PAHs and animal AAAHs. A neighbor-joining tree was generated using MEGA version X with bootstrap values from 1,000 replications. The scale bar indicates genetic distance. All other parameters were the default values. TPHs, tryptophan 5-hydroxylases; PAHs, phenylalanine 4-hydroxylases; THs, tyrosine 3-hydroxylases; AAAHs, aromatic amino acid hydroxylases. Animal AAAHs are distinctly divided into three subgroups: THs (green), PAHs (blue), and TPHs (red).

Subsequently, the *phhA* homologue was cloned from *P. fluorescens* RG11, translated it into a 263-aa protein (PfPAH), and constructed a phylogenetic tree to reflect the evolutionary separation between prokaryotic PAHs and animal aromatic amino acid hydroxylase (AAAHs). As shown in Figure 2, the tree formed two separate, well-defined clades of animal AAAHs and prokaryotic PAHs. As expected, PfPAH localized to the clade of prokaryotic PAHs. Animal AAAHs were divided into three subgroups (PAHs, TPHs, and THs), among which PAHs displayed a closer phylogenetic relationship with TPHs.

The PfPAH polypeptide was 32.81%–33.08% identical to human and rat PAH. It showed high identity to some bacterial PAHs that have been structurally characterized, including *Pseudomonas aeruginosa* (85.50%), *C. violaceum* (44.44%), and *Legionella pneumophila* (48.99%). From secondary structure prediction, the Biopterin-H domain, a signature of biopterin-dependent aromatic amino acid hydroxylases, was observed in all AAAH sequences examined. As underlined by the black line in Supplementary Figure S2, animal TPHs and PAHs harbor an N-terminal ACT (aspartate kinase–chorismate mutase–TyrA) domain that was not found in pfPAH and other bacterial PAHs. Furthermore, two His residues and a Glu residue that cooperatively bind the catalytic iron atom (closed triangles in Supplementary Figure S2), as well as the cofactor binding sites (open triangles in Supplementary Figure S2), were relatively conserved within the biopterin-H domain.

### *In Vitro* Expression and Enzymatic Analysis of PfPAH

**FIGURE 3:**
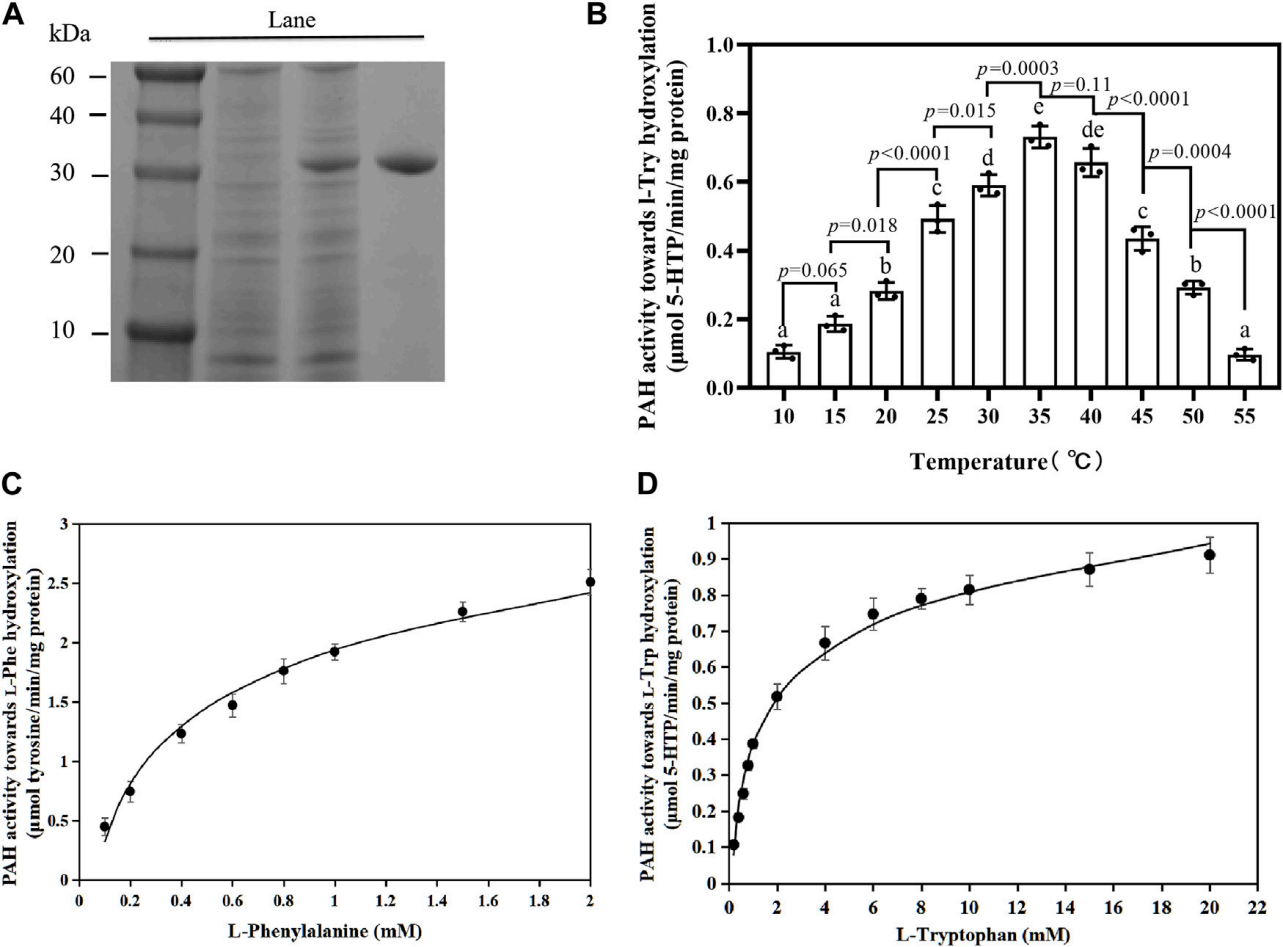
PfPAH protein characterization. **(A)** PfPAH purification in *E. coli*. SDS-PAGE image shows molecular mass markers in Lane 1, total protein of bacterial cells without IPTG in Lane 2, total protein after IPTG treatment in Lane 3, and purified PfPAH in Lane 4. **(B)** Effect of temperature on PfPAH l-Trp hydroxylase activity measured with 20 mM L-Trp, 0.5 mM BH_4_, and 100 μM FeSO_4_. Each data point represents the mean ± standard deviation (*n* = 3). Different letters indicate significant difference by Tukey’s multiple comparison test (*p* < 0.05). **(C)** Effect of l-Phe concentration on PfPAH hydroxylase activity for l-Phe. **(D)** Effect of l-Trp concentration on PfPAH hydroxylase activity for l-Trp. The curves in Figures 3C,D were fit to the non-linear Michaelis–Menten equation.

To describe the enzymatic properties of the PfPAH, a prokaryotic expression vector pET-30a-*phhA* was constructed to express recombinant PfPAH-(His)_6_ *in vitro*. After Ni^2+^ affinity chromatography and SDS-PAGE, the purified PfPAH protein had a molecular weight of ∼30 kDa (Figure 3A), and was close to its theoretical size [31.14 kDa for the PfPAH-(His)_6_].

To investigate the hydroxylase activities of PfPAH towards l-Phe and l-Trp, the reactions were performed at the optimum temperature (35°C; Figure 3B). In the hydroxylation of l-Phe, kinetic analyses showed that the *K*_m_ and *V*_max_ values were 0.73 ± 0.07 mM and 3.37 ± 0.13 μmol tyrosine/min/mg protein (Table 1; Figure 3C), respectively. However, in the hydroxylation of l-Trp, the *K*_m_ value was 1.67 ± 0.19 mM and 2.3-fold higher than that towards l-Phe; the *V*_max_ value was 0.96 ± 0.04 μmol 5-HTP/min/mg protein and 3.5-fold lower than that towards l-Phe (Table 1; Figure 3D). In addition, the catalytic efficiency (*K*_cat_/*K*_m_) of PfPAH for l-Phe hydroxylation (2.40 s^−1^ mM^−1^) was 8.0-fold higher than that for l-Trp hydroxylation (0.30 s^−1^ mM^−1^). The results indicate that PfPAH had tryptophan hydroxylase activity, but displayed lower activity and weaker substrate preference for l-Trp as compared to hydroxylate l-Phe.

**TABLE 1:** Steady-state kinetic parameters of PfPAH hydroxylase activity for amino acids.

Substrates	*V*_max_	*K*_m_ (mM)	*K*_cat_ (s^−1^)	*K*_cat_/*K*_m_ (s^−1^ mM^−1^)
l-Phenylalanine	3.37 ± 0.13 b (μmol tyrosine/min/mg protein)	0.73 ± 0.07 b	1.75 ± 0.07 a	2.40
l-Tryptophan	0.96 ± 0.04 a (μmol 5-HTP/min/mg protein)	1.67 ± 0.19 a	0.50 ± 0.02 b	0.30

Values are the mean of three replicates ± standard deviation. In columns, different letters indicate significantly different at *p* < 0.05 according to Student’s *t*-test.

### PfPAH Is Necessary for the Biosynthesis of 5-HTP and Melatonin in *P. fluorescens* RG11

PfPAH hydroxylates l-Trp into 5-HTP *in vitro*, suggesting that *phhA* may play a potential role in the biosynthesis of 5-HTP and melatonin *in vivo*. To test this functionality, a ∆*phhA* mutant RG11 strain that specifically lacks the intact *phhA* gene was generated. Notably, some melatonin precursors such as tryptamine, 5-HTP, and *N*-acetylserotonin were present in the fresh BP liquid medium at concentrations of 3.46–167.42 μg/L (Supplementary Figure S3), suggesting that these components could be present in the medium composition (beef extract or tryptone). Therefore, the *P. fluorescens* may assimilate and use these compounds in the early culture stages as melatonin intermediates. To prevent interference, ^15^N double-labeled l-Trp (^15^N-Trp) was added into the medium as the precursor. Furthermore, the flow of ^15^N-labeled tryptophan could be monitored in both wild-type and mutant strains during bacterial culture with UPLC-MS/MS analysis.

The chemical structures and chromatograms of standards (40–60 ng ml^−1^) and their corresponding ^15^N-metabolites are presented in Supplementary Figures S4A,B, respectively. As for ^15^N-5-HTP, the ^15^N-isotope increases two and one molecular mass units for the parent and daughter ions, respectively, but the retention time was identical to that of the unlabeled 5-HTP standard. So, the production of ^15^N-5-HTP was judged to have occurred when a peak was obtained at the transitions of *m/z* ^+^ 223→205. Similarly, ^15^N-serotonin, ^15^N-5-methoxytryptamine, ^15^N-*N*-acetylserotonin, and ^15^N-melatonin were all detected and confirmed in wild-type RG11 strain cultures using the process described above. However, no peak was detected at the correct retention time for ^15^N-tryptamine at the transitions of *m/z* ^+^ 163→145 throughout the experiments. These findings suggest that 5-HTP may be a key intermediate of melatonin biosynthesis in the RG11 strain.

**FIGURE 4:**
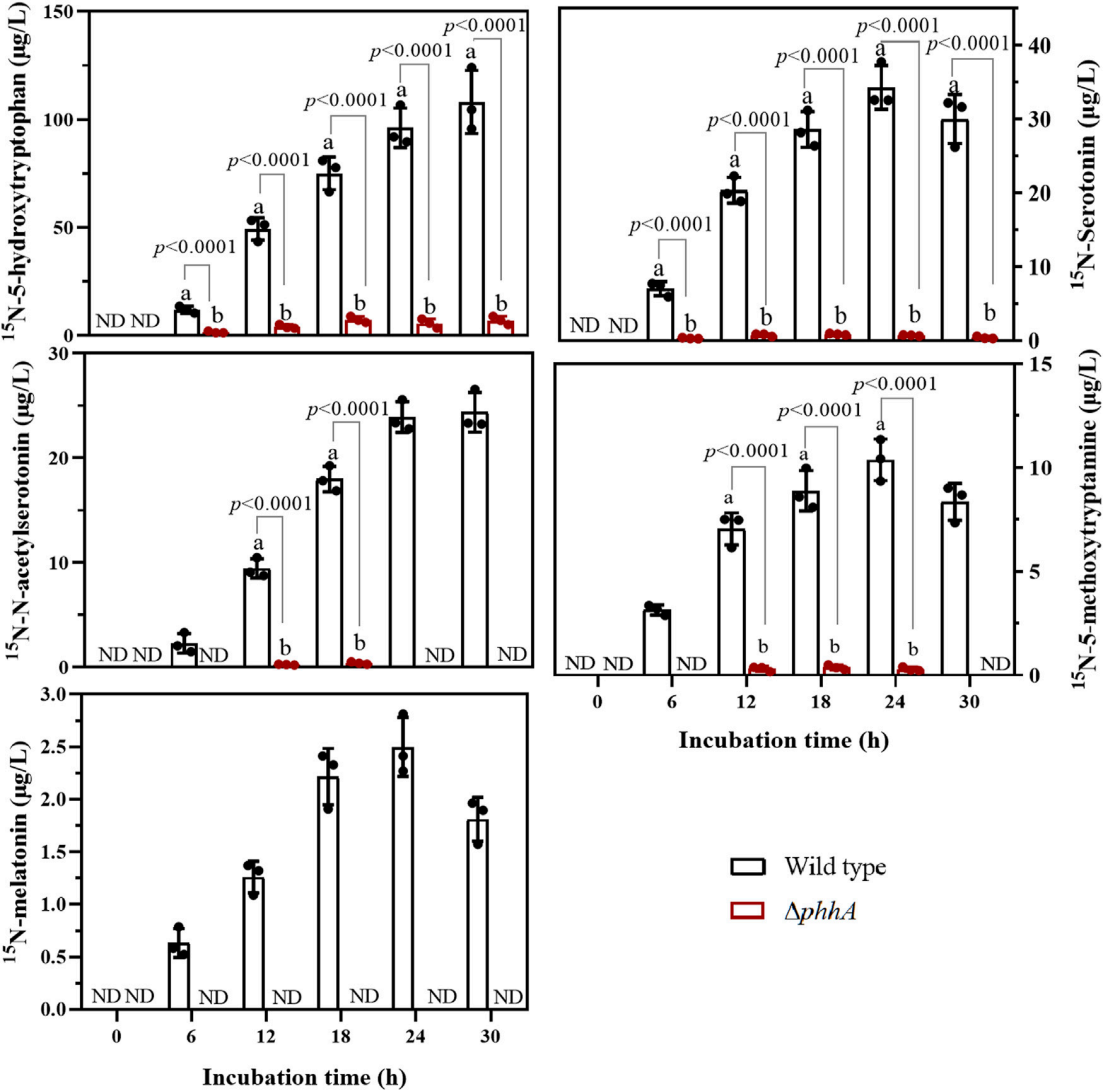
Evolution of five ^15^N-metabolites in the melatonin biosynthesis pathway in wild-type and ∆*phhA* mutant *P. fluorescens* cultures. Concentrations are expressed as μg L^−1^. Each data point represents the mean ± standard deviation (*n* = 3). Different letters indicate significant difference with the two-tailed Student’s *t*-test compared to the ∆*phhA* mutant in each time point. ND indicates this substance was not detected.

For the wild-type strain, ^15^N-5-HTP and ^15^N*-N*-acetylserotonin were detectable at 6 h and showed an increase in concentration over the course of incubation (Figure 4). Similar trends were observed for ^15^N-serotonin, ^15^N-5-methoxytryptamine, and ^15^N-melatonin, but their levels declined slightly at 30 h post-incubation. Moreover, we observed that PAH mutants displayed a significant decrease in ^15^N-5-HTP production of 1.54–7.28 μg/L (∼8- to 19-fold lower than the wild type). In addition, ^15^N-serotonin, ^15^N-5-methoxytryptamine, and ^15^N*-N*-acetylserotonin were found in small quantities at 12–30 h, but the ^15^N-melatonin was absent in ∆*phhA* mutant cultures, suggesting that PAH loss of function caused a significant decline in the levels of 5-HTP and the subsequent melatonin precursors. The findings indicate that PAH was functional in 5-HTP and melatonin biosynthesis in the RG11 strain.

### Phenylalanine 4-Hydroxylase Loss-of-Function Decreases the Tolerance to Abiotic Stress in *P. fluorescens*

**FIGURE 5:**
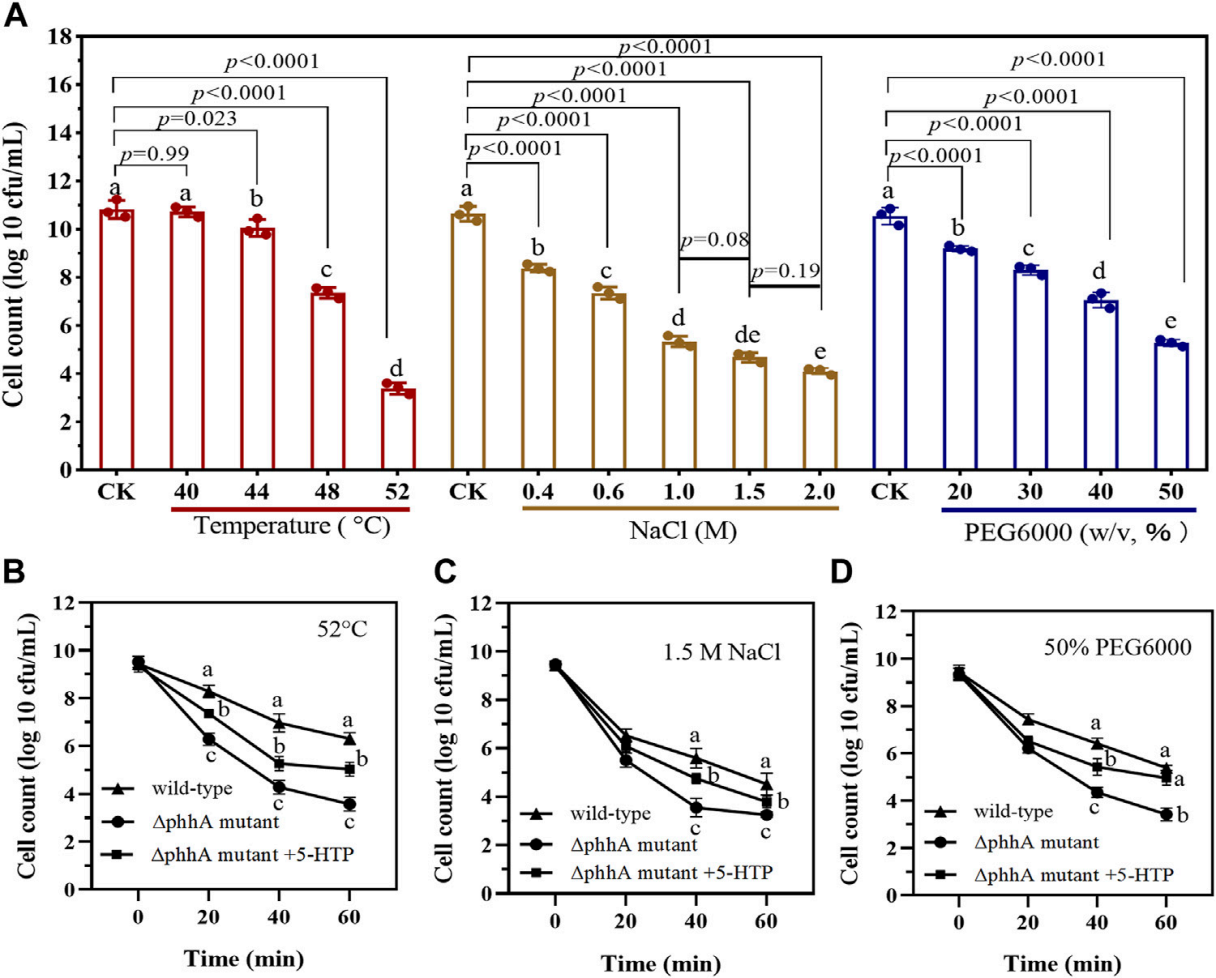
The tolerance to different abiotic stress for wild-type and ∆*phhA* mutant *P. fluorescens*. **(A)** The cell count (log number of cells per milliliter) of wild-type strain when cultured in LB medium treated with varying degrees of high temperature, NaCl, or drought stress. Cells were grown for 16 h, and the growth at each condition was monitored using the plate counting method after appropriate dilutions. **(B)** Survival (log number of cells per milliliter) of ∆*phhA* mutant strain exposed to 52°C when cultured in LB medium supplemented with 5-HTP or not. **(C)** Survival (log number of cells per milliliter) of ∆*phhA* mutant strain exposed to 1.5 M NaCl when cultured in LB medium supplemented with 5-HTP or not. **(D)** Survival (log number of cells per milliliter) of ∆*phhA* mutant strain exposed to 50% PEG6000 when cultured in LB medium supplemented with 5-HTP or not. Each data point represents the mean ± standard deviation (*n* = 3). Different letters indicate significant difference with the two-tailed Student’s *t*-test compared to the control in each treatment or time point.

The tolerance to different range of stressors for *P. fluorescens* RG11 and ∆*phhA* mutant was also evaluated. As shown in Figure 5A, wild-type RG11 strain reached the bacterial count of 10.82 log10 CFU/ml under the control condition (37°C) after incubation for 16 h. On the other hand, the overheating condition (>44°C) and the addition of salt (>400 mM) and PEG6000 (20–50%, w/v) inhibited bacterial growth. The cells of ∆*phhA* mutant strain exposed to 52°C, 1.5 M salt or 50% PEG6000 showed a significant reduction in viable cells than those of wild type strain after 20 min treatment (Figures 5B–D). Notably, an increase in survival of cells was observed when the ∆*phhA* strain was cultured under the extreme condition supplemented with 500 µg/L 5-HTP. The results suggest that the loss of function of PAH decreased the tolerance to abiotic stress in *P. fluorescens.*

## Discussion

The ability of melatonin biosynthesis may be a new biological function for endophytic bacteria in plant–microbe interactions. Several species of endophytic bacteria that synthesize melatonin were isolated previously (Jiao et al., 2016), and *P. fluorescens* RG11 is one of these endophytic bacteria and was further observed to enhance the level of endogenous melatonin in grape roots (Ma et al., 2017). However, almost all of the genes involved in the bacterial melatonin biosynthesis were not identified. The present study confirmed that PAH from *P. fluorescens* hydroxylated l-Trp to form 5-HTP and thus affected melatonin production downstream, which filled a knowledge gap in current melatonin research in microorganisms.

Phylogenomic analysis showed that animal PAHs had a closer genetic relationship with TPHs than with THs (Figure 2), suggesting that both enzymes share a higher degree of sequence similarity. According to the phylogenetic evidence and sequence homology, Lin et al. (2014) and Gjetting et al. (2001) proposed that the prokaryotic PAH gene is a progenitor of a hydroxylase that subsequently diverged to form TPHs, PAHs, and THs in animals. Thus, the substrate specificities of PAHs and TPHs are not absolute. Indeed, human TPH and PAH readily hydroxylate both tryptophan and phenylalanine with comparable kinetics (Jeffrey et al., 2001). It is reasonable to infer that prokaryotic PAHs could hydroxylate l-Trp.

Traditionally, the role of PAH in *Pseudomonas* species, such as *P. aeruginosa* and *P. putida*, is to catabolize l-Phe as a source of carbon and nitrogen (Herrera and Ramos, 2007). In the present study, PAH from *P. fluorescens* not only hydroxylated l-Phe to produce tyrosine but also hydroxylated l-Trp to form 5-HTP. The function of l-Trp hydroxylation for PAH has been verified by previous reports, in which different prokaryotic PAH-encoding genes were utilized to produce 5-HTP *via* combinatorial protein and engineered *E. coli* (Sun et al., 2015) and *Saccharomyces cerevisiae* (Zhang et al., 2016b). However, prokaryotic recombinant PAH previously displayed lower activity and affinity for l-Trp than l-Phe *in vitro* (Kino et al., 2009; Lin et al., 2014). The feature is consistent with our finding, in which the affinity (*K*_m_) of PfPAH for l-Trp was approximately 2.3-fold lower than that for l-Phe (Table 1). Additionally, PfPAH exhibited a much lower l-Trp hydroxylation activity than TPH in humans (Daubner et al., 2002). According to previous observation in *C. violaceum* (Kino et al., 2009), such l-Trp hydroxylase activity of PAH can be improved *in vitro* through mutagenesis of the amino acid sites that were expected to interact with cofactors and substrate. The l-Trp hydroxylation may be the initial step for melatonin biosynthesis in *P. fluorescens*. Therefore, it remains to be further verified whether site-specific mutagenesis of PfPAH can improve l-Trp preference and hydroxylase activity, thus enhancing 5-HTP and melatonin production.

At present, no substantial evidence exists that shows that prokaryotic PAHs is involved in bacterial melatonin biosynthesis. In the present study, ^15^N-tryptamine was not detected in the *P. fluorescens* RG11 cultures (Supplementary Figure S4), whereas the concentration of ^15^N-5-HTP increased throughout the incubation period (Figure 4). The production of ^15^N-5-HTP, as well as the subsequent production of ^15^N-serotonin, decreased to below detectability in the PAH loss-of-function strain. This suggests that serotonin was synthesized *via* l-Trp hydroxylation similar to that observed in animals or the unicellular organism *Gonyaulax polyedra* (Burkhardt and Hardeland, 1996) (Supplementary Figure S1). However, a low level of ^15^N-5-HTP was still detectable during the culture of ∆*phhA* mutant strain, indicating that the carbon skeleton of isotopic l-Trp flowed into 5-HTP by another enzyme with l-Trp hydroxylase activity, albeit operating at a lower activity. Tan et al. (2016) recently proposed an additional conversion pathway from serotonin to melatonin based on extensive evidence from plants, in which serotonin is first *O*-methylated to 5-methoxytryptamine and then *N*-acetylated to melatonin. This is because serotonin may be a more preferable substrate for plant *N*-acetylserotonin methyltransferase (ASMT), and is thus more easily *O*-methylated (Byeon et al., 2015). The preferred substrate for plant type serotonin *N*-acetyltransferases (SNATs) is 5-methoxytryptamine rather than serotonin (Lee et al., 2015). Consistently, 5-methoxytryptamine was also detected in the *P. fluorescens* RG11 strain; thus, *P. fluorescens* could produce melatonin by synthesizing 5-methoxytryptamine. The bacterial SNATs could be a member of the GCN5-related *N*-acetyltransferase (GNAT) family, which catalyzes the transfer of the acetyl group from acetyl coenzyme A to a number of compounds including histones, aminoglycosides, and arylalkylamines (Dyda et al., 2000). Given the success of identifying plant ASMT genes from the *O*-methyltransferase family of plants (Kang et al., 2011), it is fair to assume that bacterial ASMT may be discovered in the OMT family as well. Protein structure classification (Jia et al., 2021) could be used to find similar enzymes.

Overall, the study provided evidence that prokaryotic PAHs may contribute to the first step of melatonin biosynthesis in *P. fluorescens*. However, the finding does not suggest that all bacterial species rely on this protein to produce 5-HTP and melatonin. Further studies focusing on other genes, such as bacterial SNAT and ASMT, involved in the melatonin biosynthesis in *P. fluorescens* are still required. In addition, the conversion of l-Trp to tryptamine, which is one of the initial steps in melatonin biosynthesis in plants, is also an alternative pathway for indole-3-acetic acid biosynthesis in some endophytic bacteria (Hartmann et al., 1983; Carreño-Lopez et al., 2000). Therefore, it can be hypothesized that bacterial melatonin biosynthesis may be complex and species-dependent. Moreover, endophytes can interact with their plant hosts, perhaps both could synthesize melatonin by exchanging intermediates. Therefore, it is necessary to determine whether some endophytic bacteria might utilize the melatonin intermediates within plant host to produce melatonin in the symbiotic relationship.

## Data Availability

The datasets presented in this study can be found in online repositories. The names of the repository/repositories and accession number(s) can be found below: NCBI (accession: KY652930).
