# Supplementary figures and images for: Phenylalanine 4-Hydroxylase Contributes to Endophytic Bacterium Pseudomonas fluorescens’ Melatonin Biosynthesis
Source: Front Genet. 2021 Nov 15;12:746392. doi: 10.3389/fgene.2021.746392 (PMC8634680; doi:10.3389/fgene.2021.746392)

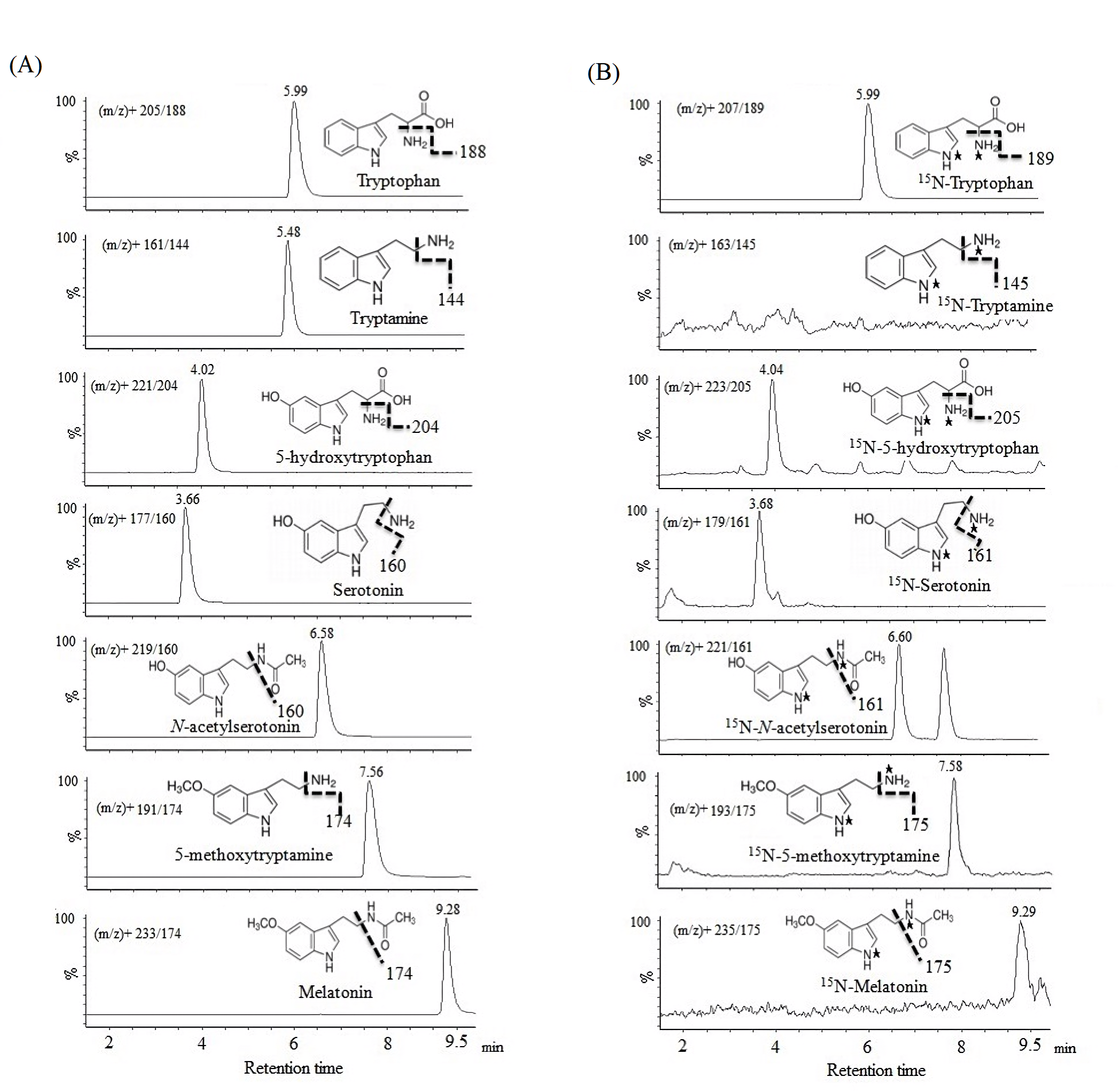

Supplement: Supplementary file 2 [file Image4.TIF]

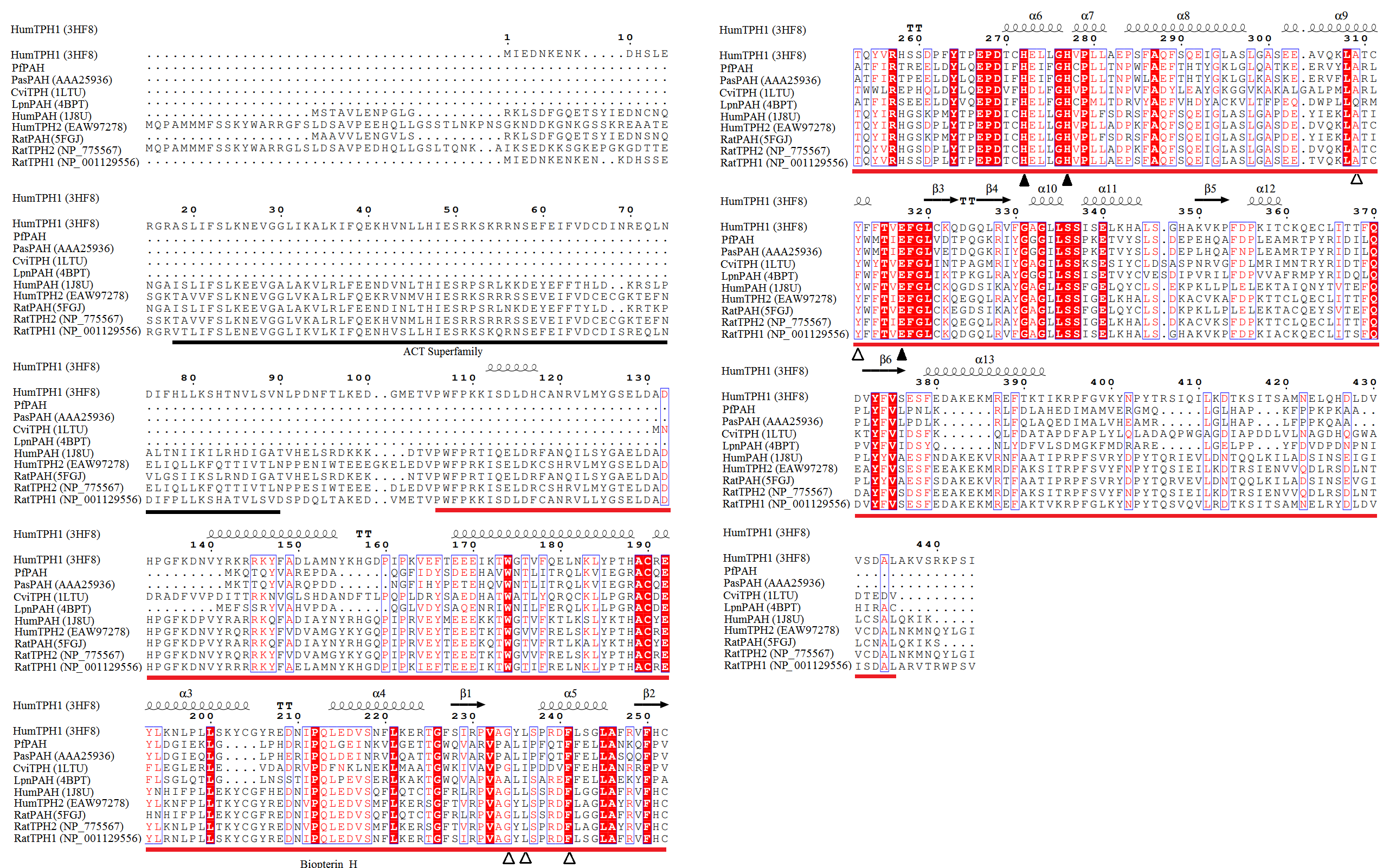

Supplement: Supplementary file 3 [file Image2.TIF]

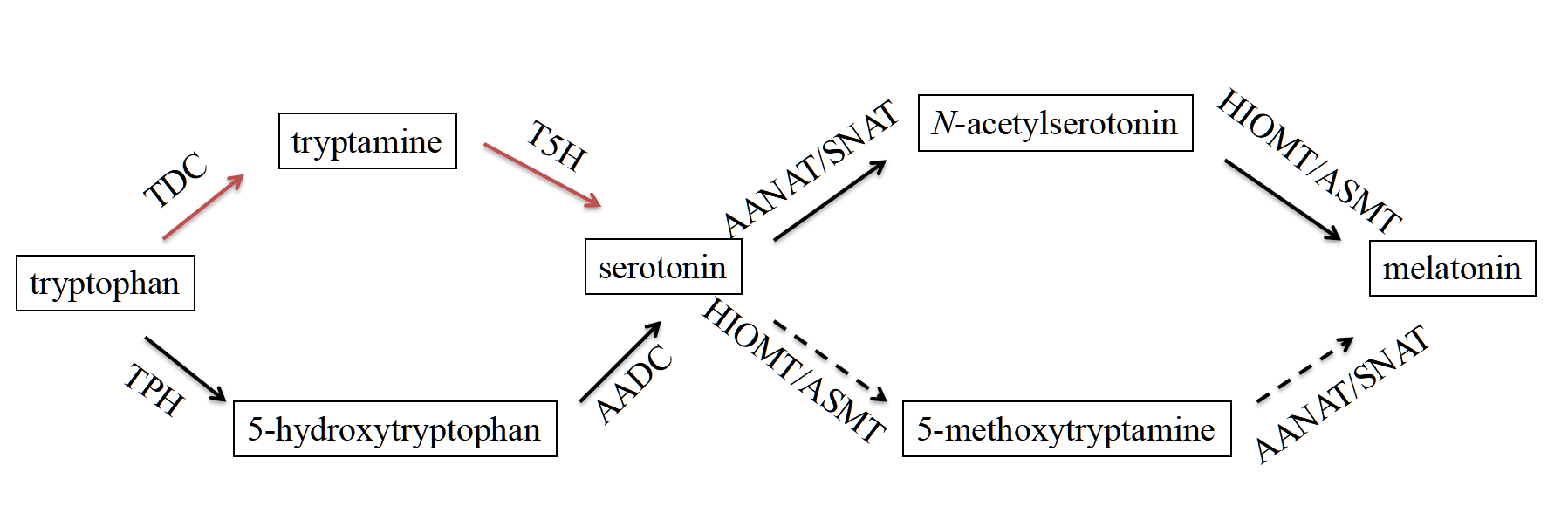

Supplement: Supplementary file 4 [file Image1.TIF]
