# Supplementary material for: Phenylalanine 4-Hydroxylase Contributes to Endophytic Bacterium Pseudomonas fluorescens’ Melatonin Biosynthesis
Source: Front Genet. 2021 Nov 15;12:746392. doi: 10.3389/fgene.2021.746392 (PMC8634680; doi:10.3389/fgene.2021.746392)
Supplement: Supplementary file 5 [file Table1.doc]

**Table S1. Strains and plasmids used in this study**

| **Strains and plasmids** | **Characteristic** | **Source or reference** |
| --- | --- | --- |
| **Strains** |  |  |
| *P. fluorescens* RG11 | *P. fluorescens* wild-type | This study |
| *E.coli* DH5α | *E. coli* cloning strain | TaKaRa |
| *E.coli* BL21 (DE3) | prokaryotic expression strain | Tiangen, Beijing, China |
| BL21-pET-30a-*phhA* | prokaryotic expression strain for *phhA* | This study |
| *∆phhA* mutant | PAH deletion mutant strain of *P. fluorescens* | This study |
| **Plasmids** |  |  |
| pK18*mobsacB* | Suicide vector derived from plasmid pK18, Mob^+^ *sacB* Km^R^ | [31] |
| pK18-∆*phhA* | Suicide vector used for PAH deletion | This study |
| pMD 18-T | Cloning vector, Amp^R^ | TaKaRa |
| pET-30a (+) | IPTG-inducible prokaryotic expression vector, Kan^R^ | Novagen |
| pET-30a-*phhA* | prokaryotic expression vector for *phhA* | This study |
| pRK600 | Broad-host-range helper vector | [32] |
