# Supplementary material for: Phenylalanine 4-Hydroxylase Contributes to Endophytic Bacterium Pseudomonas fluorescens’ Melatonin Biosynthesis
Source: Front Genet. 2021 Nov 15;12:746392. doi: 10.3389/fgene.2021.746392 (PMC8634680; doi:10.3389/fgene.2021.746392)
Supplement: Supplementary file 6 [file Table2.doc]

**Table S2. The region-specific primers used for this study**

| **Primer name** | **Prime sequence (5’-3’)** | **Annealing temperature (ºC)** |
| --- | --- | --- |
| *phhA*-F1 | GGCATCAGCCCTACCCTTTT | 58 |
| *phhA*-R1 | GCCGTCGACGAGCGAGAGGCAGGCTCTCCTGCTAGGCATTC |
| *phhA*-F2 | TGCCTCTCGCTCGTCGACGGCGAGGCCAGGTGAGTCTGTTC | 60 |
| *phhA*-R2 | AAATGCATTACTTGCGGCCC |
